# Development and verification of a prognostic model for colon cancer on pyroptosis-related genes

**DOI:** 10.3389/fgene.2022.922055

**Published:** 2022-09-30

**Authors:** Zizhen Wu, Bo Wang, Yingjiang Ye, Shan Wang, Kewei Jiang

**Affiliations:** ^1^ Department of Gastroenterological Surgery, Peking University People’s Hospital, Beijing, China; ^2^ Laboratory of Surgical Oncology, Beijing Key Laboratory of Colorectal Cancer Diagnosis and Treatment Research, Peking University People’s Hospital, Beijing, China

**Keywords:** colon cancer, bioinformatics analysis, pyroptosis, prognosis, survival

## Abstract

**Background:** Recently, the role of pyroptosis in cancer has attracted people’s attention, but its function in colon cancer remains unclear. This study aimed to construct a pyroptosis-related model that can effectively predict the prognosis of patients with colon cancer and explore the potential functions of pyroptosis-related genes.

**Methods:** We identified 40 differentially expressed PRGs between colon cancer and normal colon tissues. The model was established using the least absolute shrinkage and selection operator (LASSO) Cox regression method, and the patients were divided into high- and low-risk groups. Finally, we verified its biological function *in vitro* based on three PRGs and demonstrated discrepant expression of PRGs within colon cancer and non-tumor tissues at the protein level with immunohistochemistry.

**Results:** A pyroptosis-related prognosis model was constructed, which divided 446 patients with colon cancer into high- and low-risk groups. Kaplan–Meier analysis results showed that the survival of patients with colon cancer in the high-risk group was worse than that in the low-risk group. Finally, we also confirmed that this score is an independent prognostic factor for colon cancer progression.

**Conclusion:** In summary, the model established by three PRGs was a reliable indicator for predicting prognosis, suggesting that pyroptosis might be a noteworthy therapeutic target in CC.

## Introduction

Colon cancer (CC) is a malignant tumor that causes cancer-related death (Siegel et al., 2020). More than 1.5 million colorectal cancer cases have been recorded in America, and 104,610 patients were recorded in 2020 (Miller et al., 2019). The main reasons for this situation can be attributed to delayed diagnosis and insufficient treatment (Sanoff et al., 2008). Considering that patients with CC are in the advanced stage at the time of diagnosis, the overall survival time after the treatment of patients with CC remains very unsatisfactory. Accordingly, effective targets should be determined to improve the clinical effect of CC.

Pyroptosis, which is described as caspase-1, induces the death of monocytes infected by viruses or bacteria (Doitsh et al., 2014). The activation of inflammatory body caspase-8 is inhibited by GSDMD-dependent pyroptosis (Shi et al., 2017). Pyroptosis is also associated with digestive cancer (Gao et al., 2018; Tan et al., 2020). The expression of GSDMD is decreased in cancer cells compared to adjacent non-cancerous cells, and this promotes the proliferation of cancer cells (Fang et al., 2020). Pyroptosis is related to IL-1β and various inflammatory stimuli (Zhang et al., 2018). In addition, PD1/PDL-1 inhibitors have the same antitumor effect as pyroptosis inducer (Tang et al., 2020). Based on the existing findings, bioinformatics analysis was used to study pyroptosis-related genes and their prognostic significance in colon cancer.

In this study, we conducted a comprehensive analysis on the data of patients with colon cancer from TCGA and GEO. Then, we identified a novel pyroptosis-related survival model consisting of three genes (SLC2A3, TMPRSS11E, and UPK3B) in CC. Finally, we verified the biological functions of three PRGs *in vitro* and proved the differential expression of PRG in colon cancer and non-tumor tissues at the protein level by immunohistochemistry. In conclusion, the model combined with the clinical information may become a potential target for the treatment of CC and become an accurate prognostic monitoring instrument.

## Materials and methods

### Datasets

The gene expression level, clinical information, and copy number variation data of 446 normal human colon samples and CC samples were downloaded from The Cancer Genome Atlas (TCGA) on 30 September 2021. These 446 CC samples included 234 males and 212 females. The mean age was 67.1 (range, 17.0–82.0) years. T1 stage, T2 stage, T3 stage, and T4 stage are 11, 76, 303, and 56 cases, respectively. A total of 265 N0 stage cases, 102 N1 stage cases, and 79 N2 stage cases were present. M0 stage and M1 stage contained 385 and 61 cases, respectively. The data (GSE103479) in the validation set were downloaded from the GEO database.

### Differential expressions of pyroptosis-related genes

According to previously published articles, we screened out 52 pyroptosis-related genes (PRGs) (Doitsh et al., 2014; Man and Kanneganti, 2015; Shi et al., 2017; Wang and Yin, 2017). First, we converted the data in TCGA into fragment per kilobase million (FPKM). We identified 40 DEGs by using the “limma” package, and the identification standard was *p* < 0.05. Then, a protein–protein interaction (PPI) model was developed by the Search Tool for the Retrieval of Interacting Genes.

### Construction of the PRG prognostic model and correlation analysis

We narrowed the gene range and established the prognosis model by the LASSO Cox regression model, which showed three genes were retained, and the *λ* value was determined by the algorithm. The model score was standardized and calculated after centralizing and standardizing the data of TCGA. The model score formula was as follows: $Risk\;Score=\overset{3}{\underset{i}{\sum }}XiYi$ (X: coefficients and Y: gene expression level). The receiver operating characteristic (ROC) curve is drawn by the R package, including “time ROC,” “survival,” and “survminer.” The GSE103479 was used for further study. We organized the correlation features of CC patients in TCGA and GEO cohorts and analyzed the clinical information by Cox regression models. We separated TCGA cohort into two subgroups (|log2FC| ≥ 1, FDR <0.05). Related function analysis of differential genes between subgroups was shown by utilizing the “clusterProfiler” package.

### Cell line

The colon cancer cell line RKO was acquired from the American Type Culture Collection. RKO was passaged for not > 6 months, which was cultured in high glucose Dulbecco’s modified Eagle’s medium supplemented at 37°C in 5% CO2 with 10% FBS.

### siRNA knockdown and transfection

First, the cells were plated into six-well plates. Then, cell transfection was performed according to the instructions of LipofectamineTM 3000 (Invitrogen, New York, United States). After hatching for 6 h, the complete medium was included to the cells. The required small interfering RNA (siRNA) sequences in this study were ordered from RiboBio (Guangzhou, China).

### Cell viability assay

The cells were plated in a 96-well culture plate, and 1,000 cells were included to each well. Ten microliters of cell counting kit-8 reagent was included to each well for 3 h. After 3 h, the optical thickness of the arrangement at 450 nm was measured.

### Colony arrangement assay

Colony arrangement was utilized to distinguish the expansion capacity of cells. A total of 1,000 cells were plated into a 6-well plate and hatched them at a 37°C cell incubator with 5% CO2. After 2 weeks, 4% paraformaldehyde and crystal violet were used for fixation and dyeing. Photographs were taken by using a camera.

### Scratch assay

The cells were plated into a 6-well plate. Until approximately 90% of the cells were reached, a vertical wound was scratched in the middle of the wells. Photographs were captured at 0 and 48 h.

### RNA extraction and SYBR Green PCR Master Mix PCR analyses

TRIzol (#15596018, Thermo Fisher) was utilized for RNA extraction. The cDNAs were reverse transcribed from the aforementioned RNAs using PrimeScriptTM RT Master Mix (RR036A; Takara). Then, q-PCR was performed with 2 × Taq PCR MasterMix (KT201; TIANGEN). GAPDH was used as an endogenous control for mRNA. The 2-ΔΔCt method was used to calculate the relative quantitative value. Primer sequences for GAPDH were as follows: forward, 5′- TGA CAT CAA GAA GGT GGT GAA GCA G -3′; reverse, 5′- GTG TCG CTG TTG AAG TCA GAG GAG -3′. Primer sequences for SLC2A3 were as follows: forward, 5′- CTA CCG ACA GCC CAT CAT CAT TTCC -3′; reverse, 5′- ACA CCT GCA TCC TTG AAG ATT CCT G -3′. Primer sequences for TMPRSS11E were as follows: forward, 5′- AGG TCA GAG TCT CAG GAT CGT TGG -3′; reverse, 5′- AGG TCA GAG TCT CAG GAT CGT TGG -3′. Primer sequences for UPK3B were as follows: forward, 5′- GCC CTA CAC ACC ACA GAT AAC AGC -3′; reverse, 5′- GGC AAG CCC ATC GAA GAC ACA G -3′.

### Immunohistochemistry demonstrates the expression of PRGs at the protein level

We demonstrated the difference in their protein level expression of SLC2A3, TMPRSS11E, and UPK3B in colon cancer and normal intestinal tissues. Immunohistochemical information was obtained from the Human Protein Atlas (http://www.proteinatlas.org/humanproteome/pathology).

### Statistical analysis

The amount of sample expressions was compared by one-way investigation of change. The Kaplan–Meier strategy was utilized to analyze the overall survival time. Statistical examinations were demonstrated by using R software (v4.1.1).

## Results

### Identification of differentially expressed genes

**FIGURE 1:**
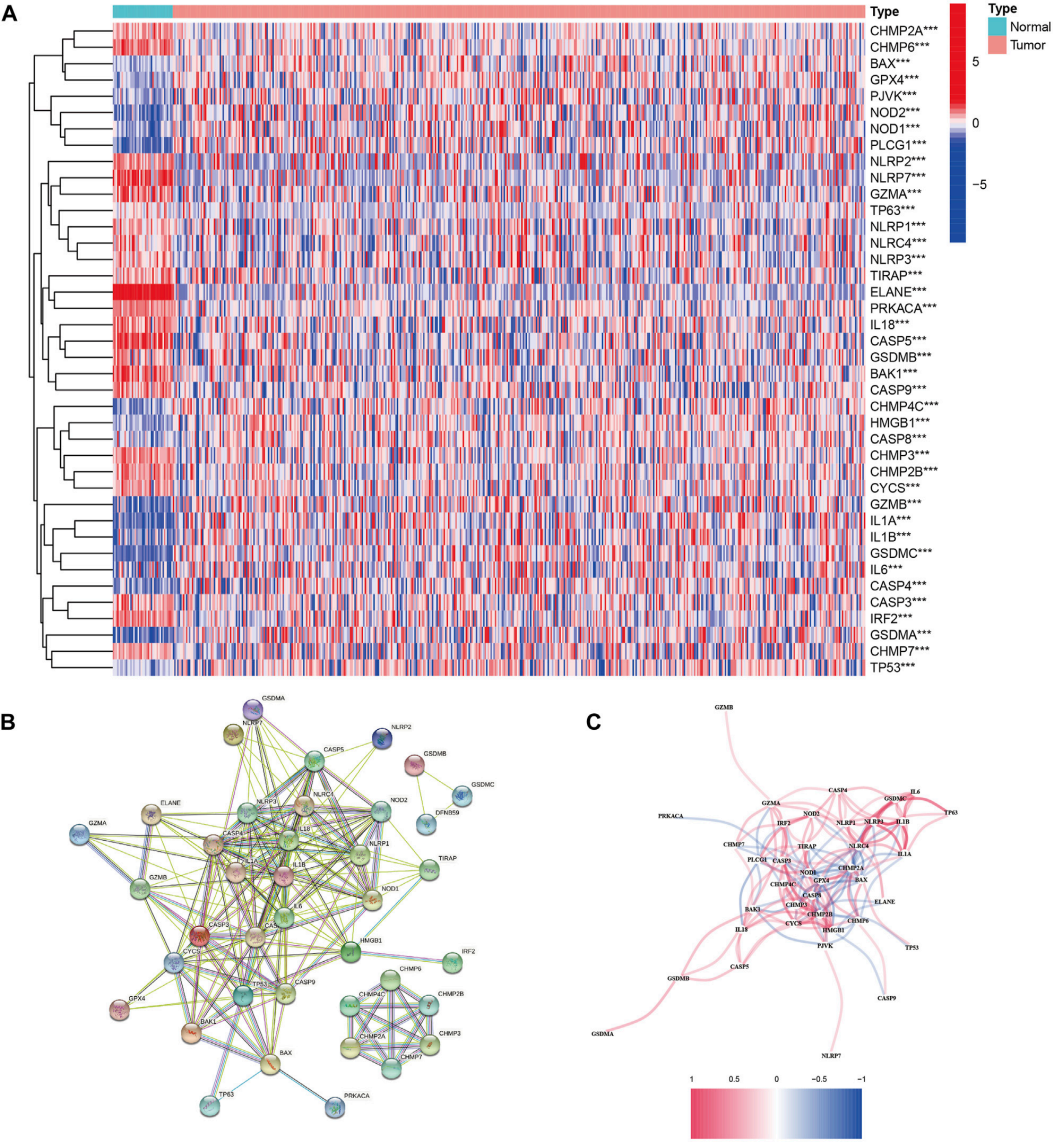
Expressions of the 40 differentially expressed genes in all 52 pyroptosis-related genes and the interactions among them. **(A)** Heatmap of the pyroptosis-related genes between the normal and tumor tissues. *p* values were shown as **p < 0.01; ***p < 0.001. **(B)** PPI network showing the interactions of the pyroptosis-related genes (interaction score = 0.9). **(C)** Correlation network of the pyroptosis-related genes (red line: positive correlation and blue line: negative correlation).

By comparing the expression of 52 PRGs in 41 normal and 473 tumor tissues in TCGA, 40 DEGs were identified. In these DEGs, 22 genes (ELANE, CASP5, NLRP7, GZMA, IL18, BAK1, CHMP6, CYCS, PRKACA, CHMP2B, IRF2, CHMP2A, CHMP3, GSDMB, CASP9, NLRC4, CASP3, NLRP3, CHMP7, TIRAP, NLRP1, and NLRP2) were downregulated, whereas 18 genes (CASP8, TP63, BAX, NOD1, GPX4, CASP4, HMGB1, TP53, PJVK, CHMP4C, IL6, PLCG1, NOD2, IL1B, GZMB, GSDMA, GSDMC, and IL1A) were upregulated (Figure 1A and Supplementary Figure S1A). To investigate the interactions of these PRG, we set the minimum interaction score for protein–protein interaction (PPI) analysis to 0.5 (Figure 1B). The relationship arrangement containing all PRGs is presented in Figure 1C.

### Landscape of the genetic variation of PRGs

**FIGURE 2:**
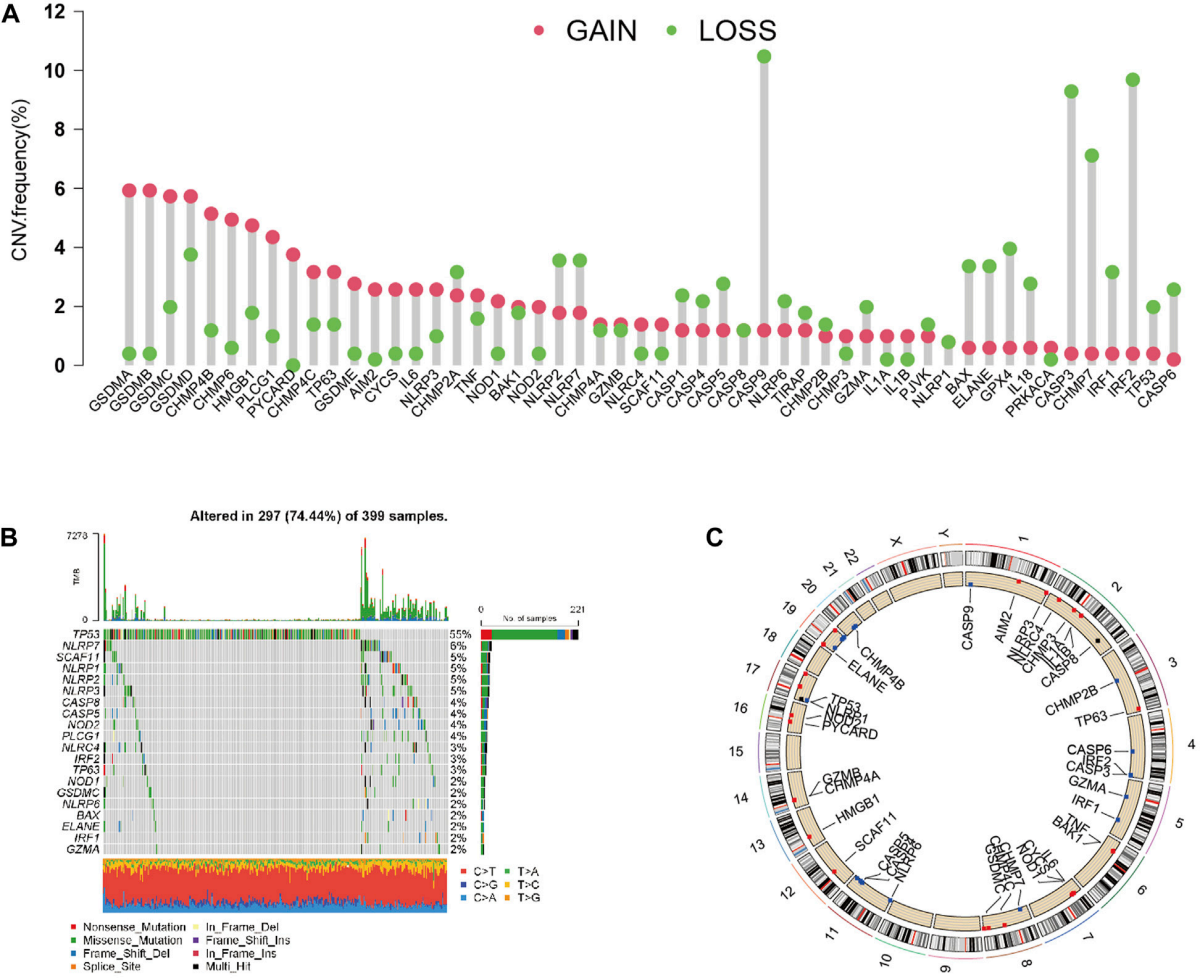
Landscape of genetic and expression variation of PRGs in CC. **(A)** CNV variation frequency of 33 PRGs in the CC cohort. The height of the column represented the alteration frequency. **(B)** Waterfall plots of mutation information in CC. **(C)** Location of CNV alteration of PRGs on 23 chromosomes in the CC cohort.

After investigating the alteration frequency of CNV, we found that all PRGs showed CNV changes. Most of the genes were widespread by the copy number, including CHMP2A, CASP1, CASP4, CASP5, CASP8, CASP9, NLRP6, TIRAP, CHMP2B, GZMA, PJVK, NLRP1, BAX, ELANE, GPX4, IL18, CASP3, CHMP7, IRF2, TP53, CASP6, and GPX4 (Figure 2A). At that point, we summarized the rate of CNV and somatic mutations of 52 genes in CC. As shown in Figure 2B, 297 of 399 (74.44%) CC samples demonstrated genetic mutations. The mutation frequencies from high to low were TP53, NLRP7, and SCAF11. Figure 2C shows the area of CNV changes of PRGs.

### Related function analysis of PRGs

Through GO analysis, we demonstrated that 52 PRGs were involved in interleukin-1 beta production, pyroptosis, and endopeptidase activity (Supplementary Figure S1B). Through KEGG analysis, we demonstrated that 52 PRGs were included in various signal pathways, such as pyroptosis (Supplementary Figure S1C).

### Tumor classification based on the DEGs

**FIGURE 3:**
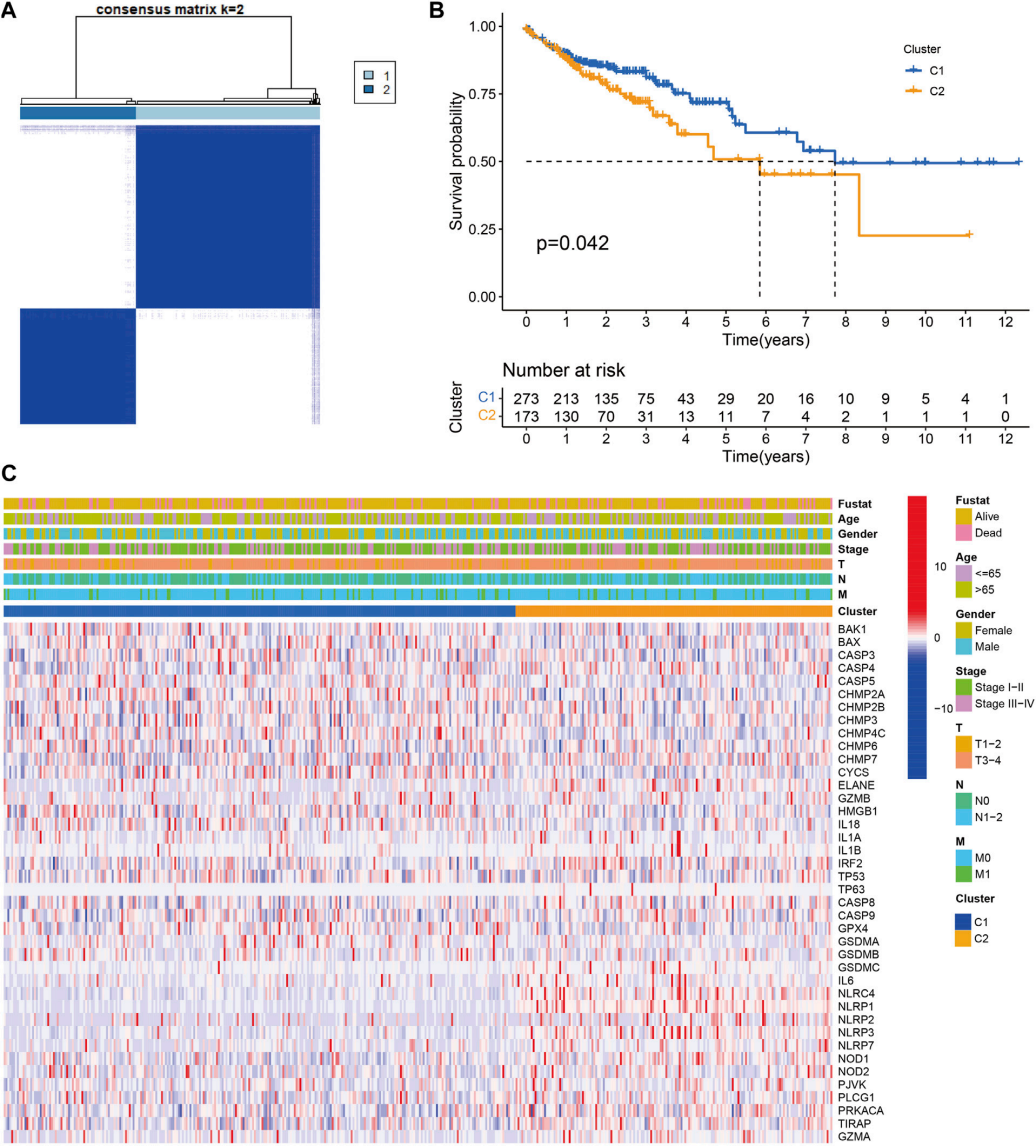
Tumor classification based on the pyroptosis-related DEGs. **(A)** 446 CC patients were grouped into two clusters according to the consensus clustering matrix (k = 2). **(B)** Kaplan‐Meier OS curves for the two clusters.**(C)** Heatmap and the clinicopathologic characters of the two clusters classified by these DEGs

**FIGURE 4:**
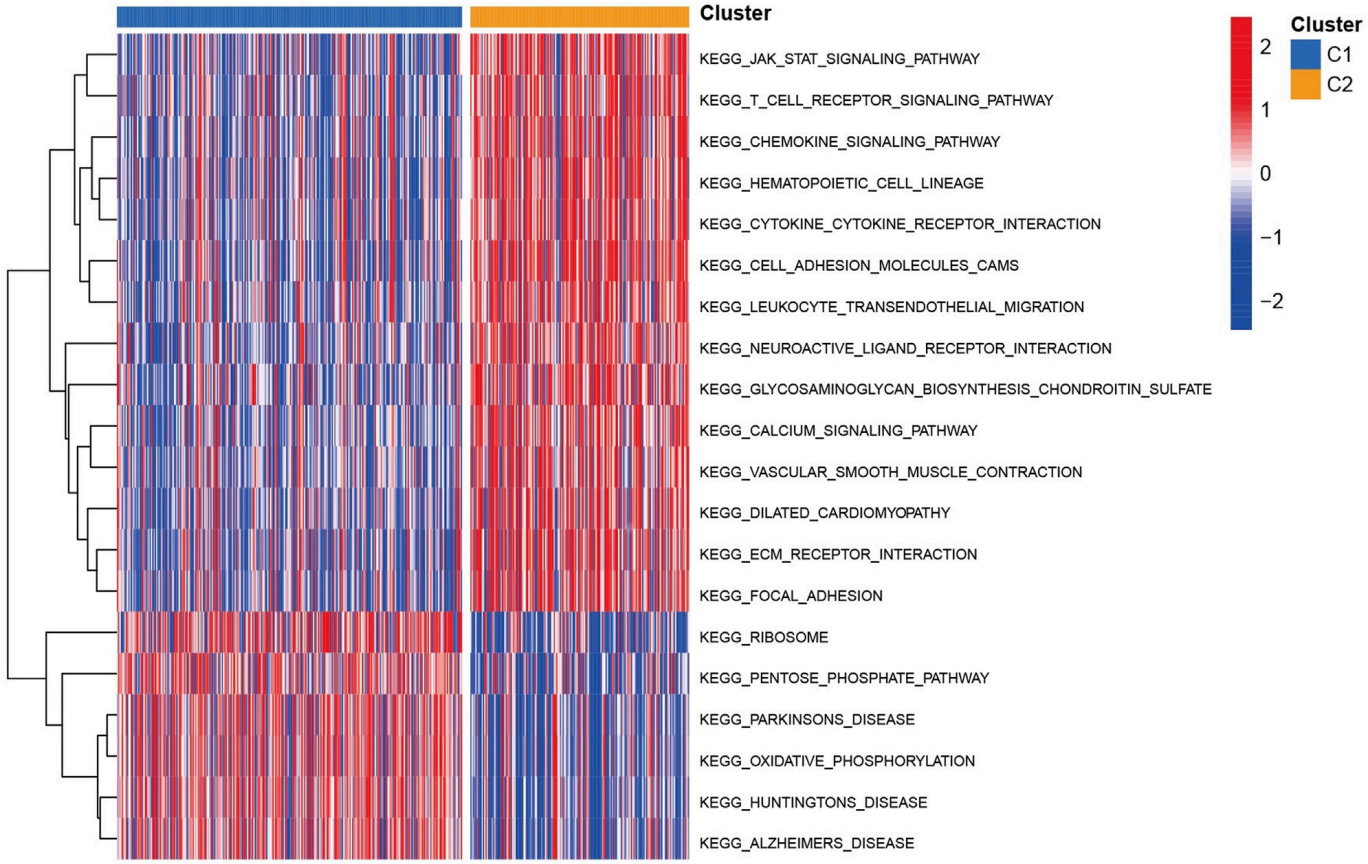
KEGG pathway enrichment analysis between two cluster groups in TCGA cohort.

A total of 446 CC samples, which were matched with corresponding complete clinical information, were included in our research. To investigate the CC subtypes among the 40 DEGs, we performed a consensus cluster with 446 CC patients. By expending the clustering value (*k*) from 2 to 9, the relationship was higher within the group and lower between groups when NK = 2, indicating that 446 sets were separated into two sets (Figure 3A). A noteworthy distinction was observed in the overall survival (OS) time (*p* = 0.042, Figure 3B). The amount of PRGs and the related clinical information included the survival status, age, gender, stage, T stage, N stage, and M stage (Figure 3C). Hence, we connected GSVA enrichment examination to investigate the biological behavior between different gene clusters. Noteworthy PRG cluster C2 enrichment in pathways related to immune cell activation was observed in various pathways, such as the T cell receptor signaling pathway (Figure 4).

### Construction of a prognostic model in TCGA

**FIGURE 5:**
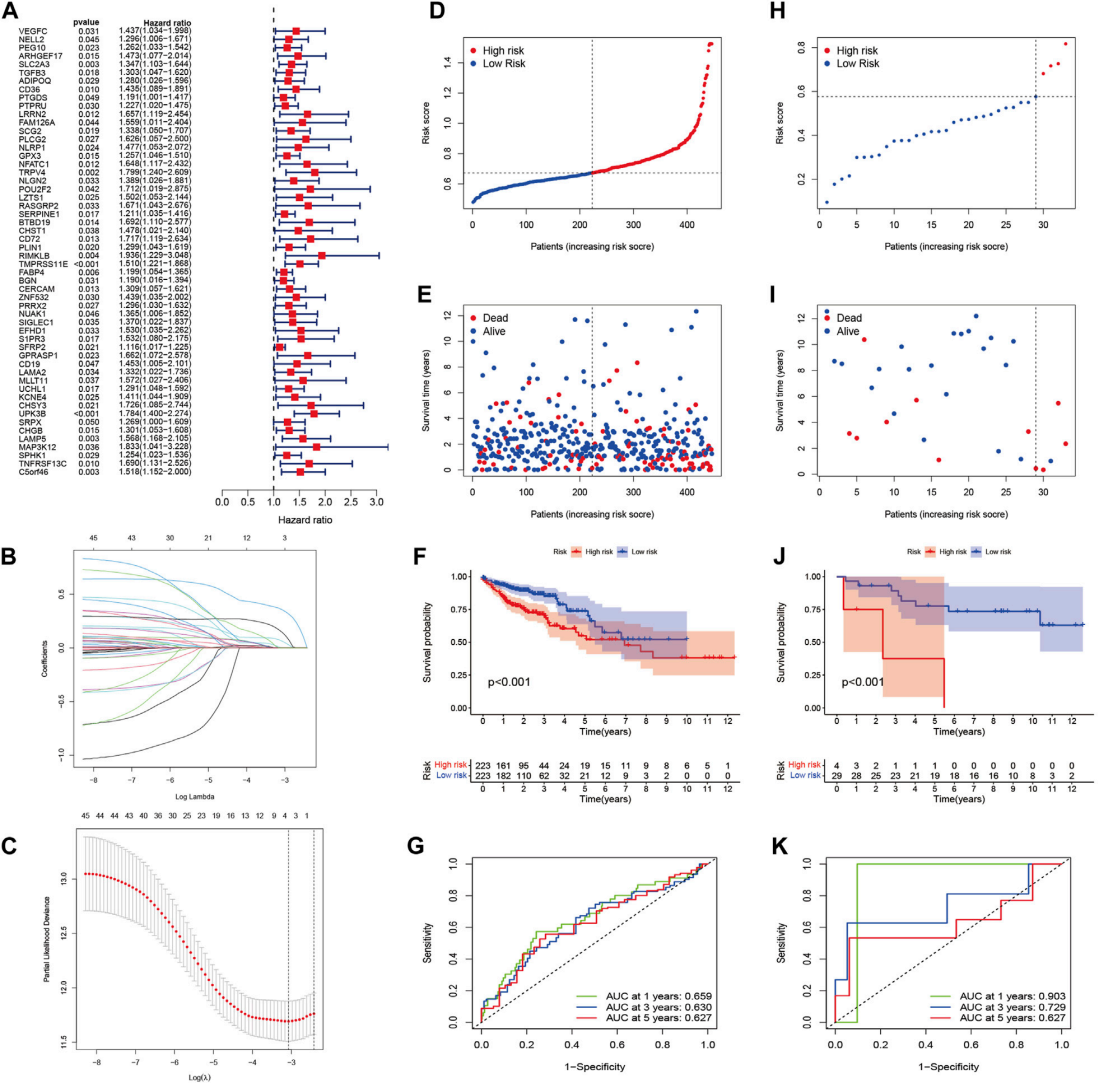
Construction of the risk signature in the TCGA cohort and verification of the model in the GEO cohort (GSE103479). **(A)** Univariate Cox regression analysis of OS for each pyroptosis-related gene and 54 genes with *p* < 0.001. **(B)** LASSO regression of the three OS-related genes. **(C)** Cross-validation for tuning the parameter selection in the LASSO regression. **(D)** Distribution of patients based on the risk score in the TCGA cohort. **(E)** Survival status for each patient in the TCGA cohort. **(F)** Kaplan–Meier curves for comparison of the OS between low- and high-risk groups in the TCGA cohort. **(G)** Time-dependent ROC curves for OCs in the TCGA cohort. **(H)** Distribution of patients based on the risk score in the GEO cohort. **(I)** Survival status for each patient in the GEO cohort. **(J)** Kaplan–Meier curves for comparison of the OS between low- and high-risk groups in the GEO cohort. **(K)** Time-dependent ROC curves for OCs in the GEO cohort.

Univariate Cox relapse examination was carried out for the essential screening of the survival-related sets. A total of 54 qualities that met the criteria of *p* < 0.05 were held for advanced investigation and were associated with HRs >1 (Figure 5A). Through LASSO Cox relapse analysis, three genes were built agreeing to the optimum *λ* esteem (Figures 5B,C). The score was calculated using the formula: Risk score = (0.0629) × SLC2A3 + (0.1449) × TMPRSS11E + (−0.3557) × UPK3B. We divided 446 patients into low- and high-risk sets concurring to the middle of the scoring formula (Figure 5D). High-risk sets had a shorter survival time than low-risk sets (Figure 5E). A noteworthy distinction was observed in the OS time between the two sets (*p* < 0.001, Figure 5F). By using ROC investigation to assess the affectability and specificity of the model, we demonstrated that the 1-, 3-, and 5-year survival rates under the ROC curve were 0.659, 0.630, and 0.627, respectively (Figure 5G).

### External validation of the risk model

A total of 33 patients in GSE103479 were used as a validation set. Before analysis, we standardized quality expression information by “scale” work. According to the median risk of TCGA, 29 sets within the GEO were grouped into the low-risk set, whereas the four other sets were grouped into the high-risk set (Figure 5H). Figure 5I demonstrates that the high-risk patients with CC had poor prognosis. In expansion, Kaplan–Meier examination analysis also demonstrated noteworthy differences between the two sets (*p* < 0.001, Figure 5J). The ROC curve demonstrated that the GEO cohort, as a validation cohort, has a high expectation effect (AUC = 0.903, 0.729, and 0.627 for 1, 3, and 5-years survival, respectively) (Figure 5K).

### Gene mutations

**FIGURE 6:**
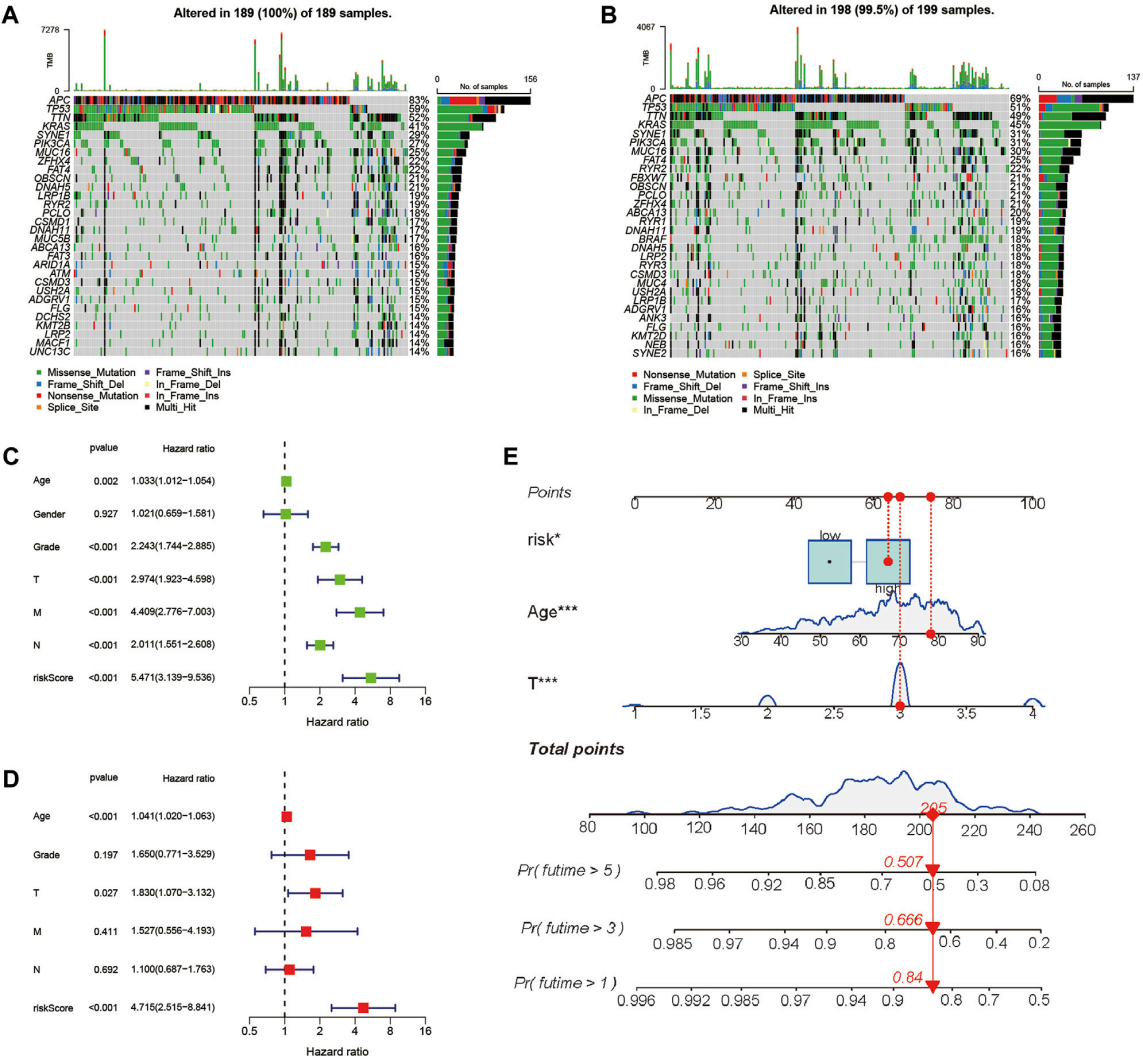
Landscape of mutation profiles between high- and low-risk CC patients and evaluation of the prognostic role of the PRG signature. **(A,B)** Waterfall plots of mutation information in each sample. **(C)** Univariate Cox forest map of the risk model score and clinical features in the TCGA cohort. **(D)** Multivariate Cox forest plot of the risk model score and clinical characteristics in the TCGA cohort. **(E)** Establishment of a nomogram for 1-, 3-, and 5-year OS prediction in CC.

We investigated the gene mutation profiles of 446 CC patients in TCGA. These two groups consisted of 189 (42.4%) and 198 (44.4%) samples, individually, while 59 (13.2%) sets were removed because of the lack of mutation information. Waterfall plots were used to assess the genes of the patients within the two sets (Figures 6A,B). The top two mutated genes were APC and TP53. Most of the mutations in the two groups were missense mutations. C-to-T transversions account for the majority single-nucleotide variation. The interaction between mutant genes is demonstrated (Supplementary Figures S2A–D).

### Prognostic correlation analysis of the risk model

**FIGURE 7:**
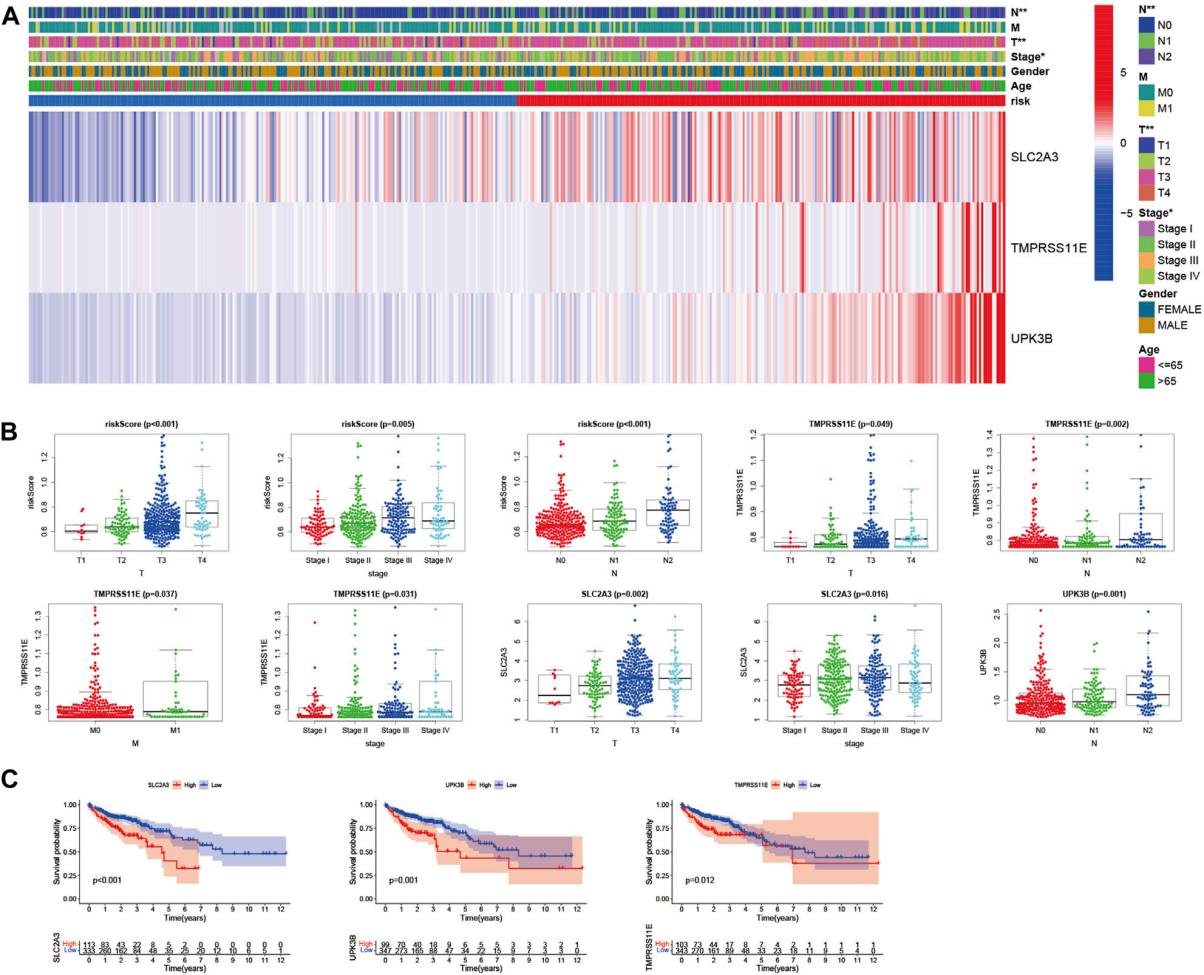
Evaluation of the prognostic model with clinical characteristics in colon cancer. **(A)** Heatmap for the connections between clinical characteristics and the risk groups (*p < 0.05, **p < 0.01). **(B)** Distributions of the risk score in CC patients with different clinical characteristics. **(C)** Kaplan–Meier survival curves according to the mRNA expression of SLC2A3, TMPRSS11E, and UPK3B in CC tissues.

To evaluate whether the score of this model can be used as a free prognostic figure, we used univariate and multivariate Cox relapse investigation to assess the hazard of quality signature demonstrate. After univariate cox relapse examination, we found that the hazard score was an independent risk factor for predicting poor survival (HR = 5.471, 95% CI: 3.139–9.536, Figure 6C). Multivariate cox relapse investigation results show that the score was an independent risk factor for predicting poor survival (HR = 4.715, 95% CI: 2.515–8.841, Figure 6D). The clinical characteristics and pyroptosis-related prognostic characteristics were included in the hybrid nomogram, indicating that it can be used for the treatment of patients with CC (Figure 6E). We further analyzed the clinical characteristic heatmap of TCGA cohort and found differences in the distribution of the stage, T stage, and N stage between subgroups (*p* < 0.05, Figure 7A). Then, we investigated the scores and mRNA expression levels of the three genes, according to clinical parameters (Figure 7B). Kaplan–Meier analysis presented that patients with CC having high SLC2A3, UPK3B, and TMPRSS11E mRNA expressions had poor survival. To test the utility of the prognostic signature, we also analyzed the age, gender, stage, T stage, N stage, and M stage (Supplementary Figure S3).

### SLC2A3, TMPRSS11E, and UPK3B enhance the proliferation and migration of colon cells

**FIGURE 8:**
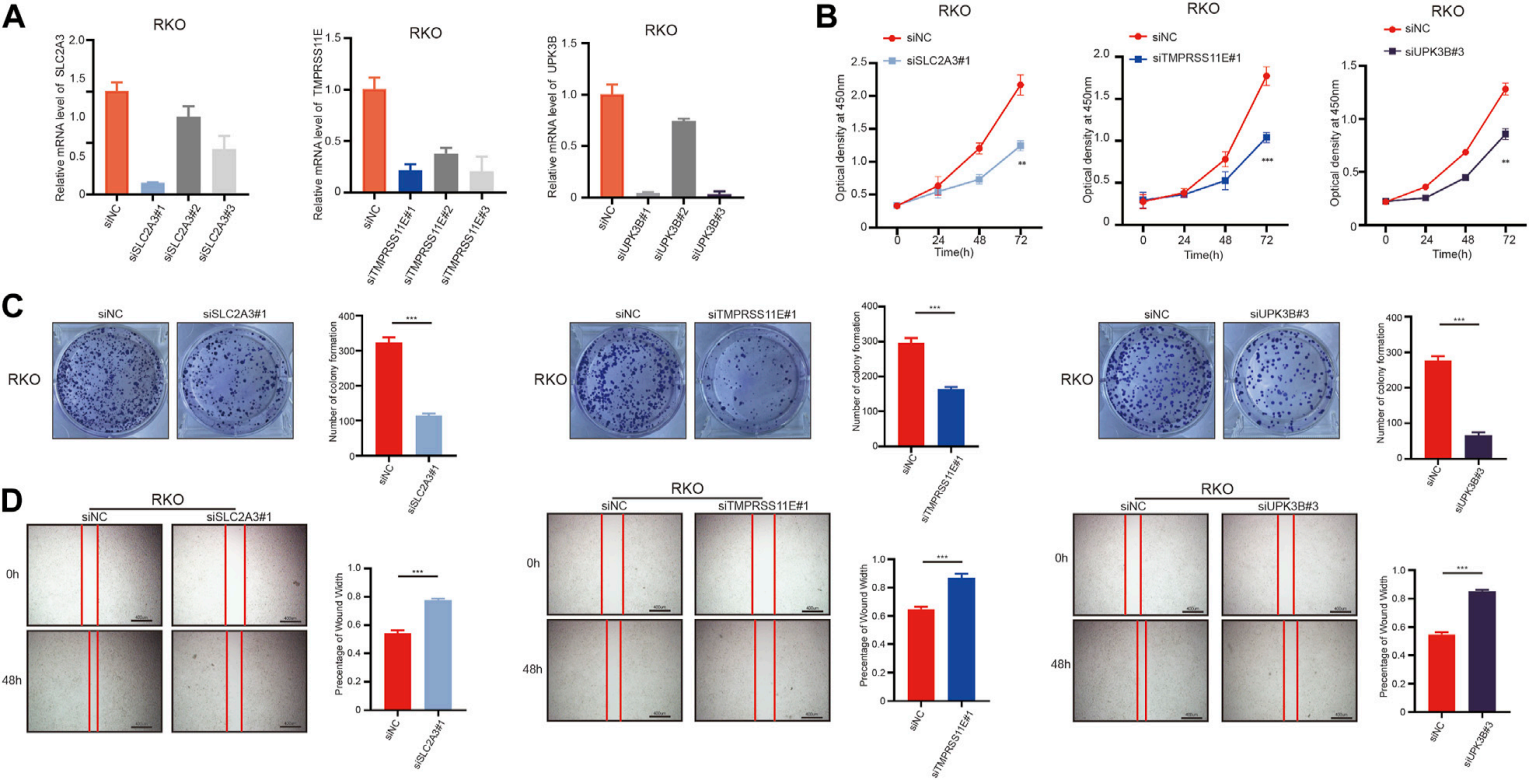
SLC2A3, TMPRSS11E, and UPK3B activities are required for tumor cell proliferation and migration. RKO cells were treated with either a control siRNA or a siRNA targeting SLC2A3, TMPRSS11E, and UPK3B. Transduced cells were used for the analysis of mRNA expression by RT-q-PCR and cell function. **(A)** mRNA levels in these established cell lines were verified by RT-q-PCR assay. **(B)** CCK-8. **(C)** Colony formation assay. **(D)** Cell-based scratch assay.

To investigate the biological function of SLC2A3, TMPRSS11E, and UPK3B in colon cells, we used siRNA to transfect RKO cells and downregulate SLC2A3, TMPRSS11E, and UPK3B. The siRNA NC was also transfected into RKO cells (Figure 8A). The cell increases the curve of the CCK-8 measures appeared that the downregulation of SLC2A3, TMPRSS11E, and UPK3B hindered cell expansion (Figure 8B). Essentially, less colonies were shaped within the downregulated SLC2A3, TMPRSS11E, and UPK3B groups than within the control set (Figure 8C). Consequently, cell viability was weak within the downregulated SLC2A3, TMPRSS11E, and UPK3B in RKO cells. Wound healing measures were investigated to examine the effect of SLC2A3, TMPRSS11E, and UPK3B on the migration capacity of RKO cells (Figure 8D). The results showed that the downregulation of SLC2A3, TMPRSS11E, and UPK3B could reduce the cell proliferation and migration of RKO cells.

### Immunohistochemistry demonstrates the protein level of PRGs

**FIGURE 9:**
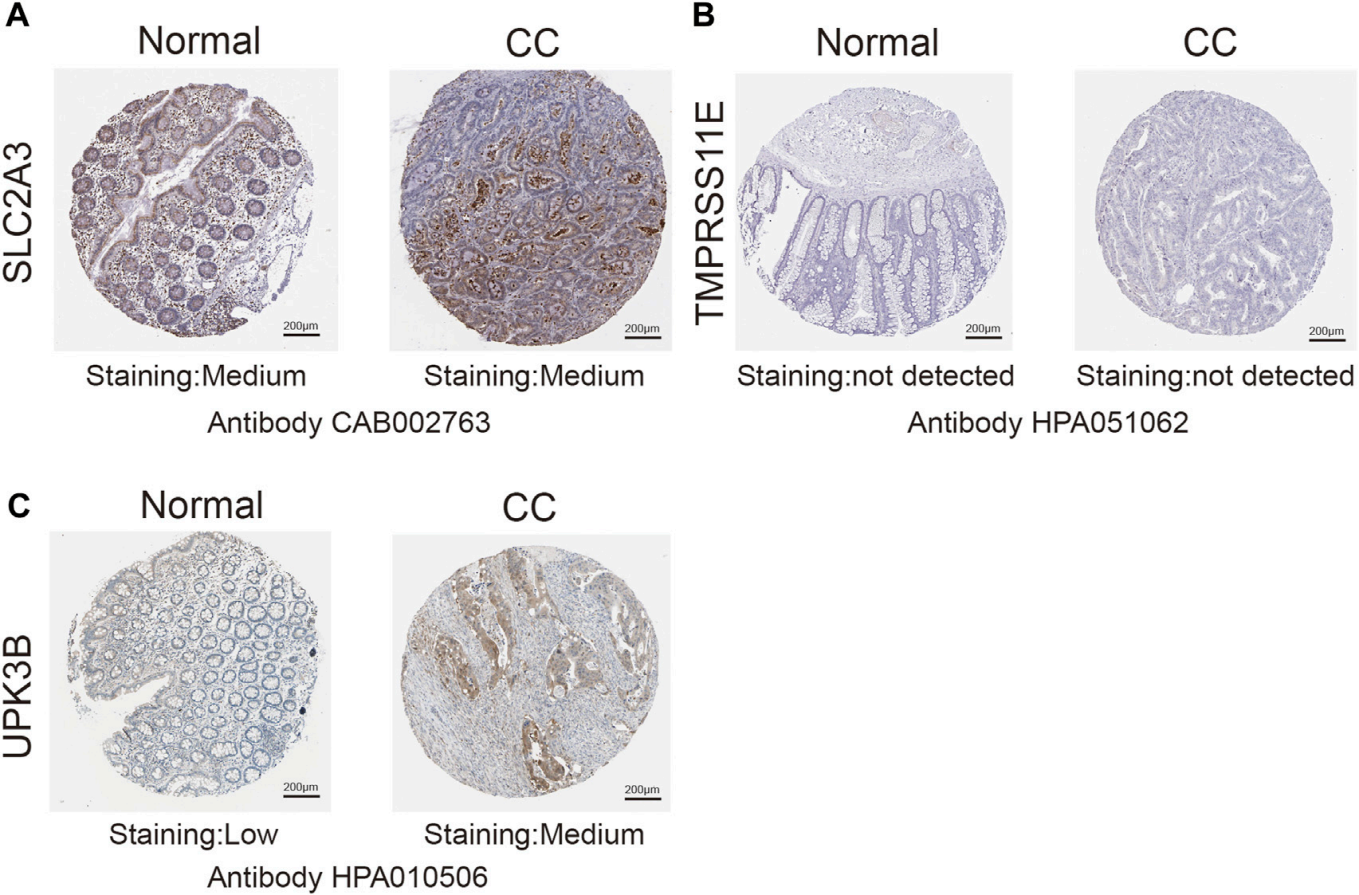
Immunohistochemistry (IHC) results showing protein levels of PRGs in CC and normal tissues. **(A)** IHC results of SLC2A3 in normal tissue and in CC. **(B)** IHC results of TMPRSS11E in normal tissue and in CC. **(C)** IHC results of UPK3B in normal tissue and in CC.

Immunohistochemical methods were used to compare the expression of PRGs (SLC2A3, TMPRSS11E, and UPK3b) in colon cancer and their expression in normal gastric tissues (Figure 9). Inevitably, the protein expression levels of these two high-risk genes (SLC2A3 and UPK3b) were significantly increased in tumor samples, with more obvious antibody staining and higher percentage of stained tissues.

## Discussion

The clinical stages of colon cancer are Tis (Tumor *in situ*), I stage, II stage, III stage, and IV stage. When the disease develops to IV stage, the 5-year survival rate is less than 30%. Surgery combined with chemoradiotherapy is effective for the treatment of early colon cancer (Tariq et al., 2016). However, some patients usually miss the best time for treatment because they ignore the symptoms. The prognosis of advanced colon cancer is poor, and the recurrence rate is high. The potential prognostic biomarkers of colon cancer should be determined. Many studies have identified biomarkers through database mining, which can analyze the prognosis of patients with cancer individually. Pyroptosis is a popular type of cell death. Pyroptosis can play a dual role in the occurrence and development of tumors. For instance, ordinary cells are fortified by an expansive number of provocative components discharged by pyroptosis (Karki and Kanneganti, 2019). Moreover, promoting tumor cell pyroptosis is expected to become a new therapeutic target for tumor patients (Xia et al., 2019). During the development of cancer, considering the high methylation of the promoter of pyroptosis-related genes, the expression level of GSDM decreases, resulting in tumor growth and metastasis (Garg and Agostinis, 2017). In the present study, TCGA data showed the mRNA expression level of 52 known coke death-related genes, and 40 of these PRGs were differentially expressed. In terms of clinical characteristics, the two clusters generated by cluster analysis based on the PRGs showed significant differences. The prognostic esteem of patients with CC was determined by developing the PRG model. The ROC curve demonstrated that the AUC of TCGA at 3 years constructed by three PRGs was 0.630, and the AUC of GSE103479 at 3 years constructed by three PRGs was 0.729. Our results demonstrated that the model constructed by three PRGs is better than that from the current guidelines in prediction. In conclusion, the risk score we established using three PRGs was a reliability index for the prediction of colon cancer. Based on our analysis, we developed a model that includes three PRGs (SLC2A3, TMPRSS11E, and UPK3B). The SLC2A family mainly focuses on the transmembrane transport of nutrients, such as glucose, which contains 14 sets from SLC2A1–14 (César-Razquin et al., 2015). The expression of the SLC2A family is different in cancer, and the amount of SLC2A1 is enhanced in CC, which can predict poor prognosis and clinical characteristics (Medina and Owen, 2002). These members of the SLC2A family have tissue-specific expression. SLC2A1 is widely expressed, and SLC2A2 and SLC2A3 are usually expressed in liver and brain tissues (Jun et al., 2011). TMPRSS113 is related to the occurrence of esophageal squamous cell carcinoma, making the cells sensitive to apoptosis under the stimulation of apoptosis (Ng et al., 2016). UPK3B, one of the significant structural components of urothelial tissue, is a member of the urothelial family (Yu et al., 1990). UPK3B is a marker of the mesothelial lineage (López-Ríos et al., 2006). The knockdown of these three genes significantly represses the proliferation and migration of colon cancer cells. The molecular effect of three PRGs is still insufficiently understood in colon cancer, and examination of the basic instruments is needed. Thus, we built up a nomogram to instinctively calculate OS. Then, the prognostic value of the PRGs was discovered *via* Kaplan–Meier survival investigation. In addition, focusing on the examination of particular histological sets of CC patients will raise the specificity and personality of PRGs. We then analyzed and verified PRGs in patients with CC having clinical characteristics and molecular signatures. To date, precision genomic medicine mainly aims to discover the precise and particular indicators of survival and prognosis (Bian et al., 2019). Recent studies have aimed to use bioinformatics investigation to determine the prognostic factors related to pyroptosis (Jiang et al., 2021; Liu et al., 2021; Shao et al., 2021; Wu et al., 2021).

In our study, there are also some limitations. Although we use multiple databases, all the data are retrospective and lack data from our center for further verification. Therefore, prospective cohort studies of CC patients receiving immunotherapy are needed to validate these findings. In addition, not all CC patients will benefit from this model. Our study can only provide preliminary theoretical support for future experimental verification.

## Conclusion

In the present study, three genes were identified that can affect pyroptosis. Our research produced a signature highlighting three PRGs (SLC2A3, TMPRSS11E, and UPK3B) and found that it can predict OS in patients with CC. We also analyzed the prognostic value of these three genes and preliminarily studied the phenotype *in vitro*. The results can be further verified by further studying the three PRGs in terms of molecular mechanism. Then, we identified a novel pyroptosis-related survival model consisting of three genes, namely, SLC2A3, TMPRSS11E, and UPK3B, in CC. In conclusion, the model combined with the clinical information may become a potential target for the treatment of CC and become an accurate prognostic monitoring instrument.

## Data Availability

The original contributions presented in the study are included in the article/Supplementary Material; further inquiries can be directed to the corresponding author.
