# Supplementary figures and images for: Development and verification of a prognostic model for colon cancer on pyroptosis-related genes
Source: Front Genet. 2022 Sep 30;13:922055. doi: 10.3389/fgene.2022.922055 (PMC9562195; doi:10.3389/fgene.2022.922055)

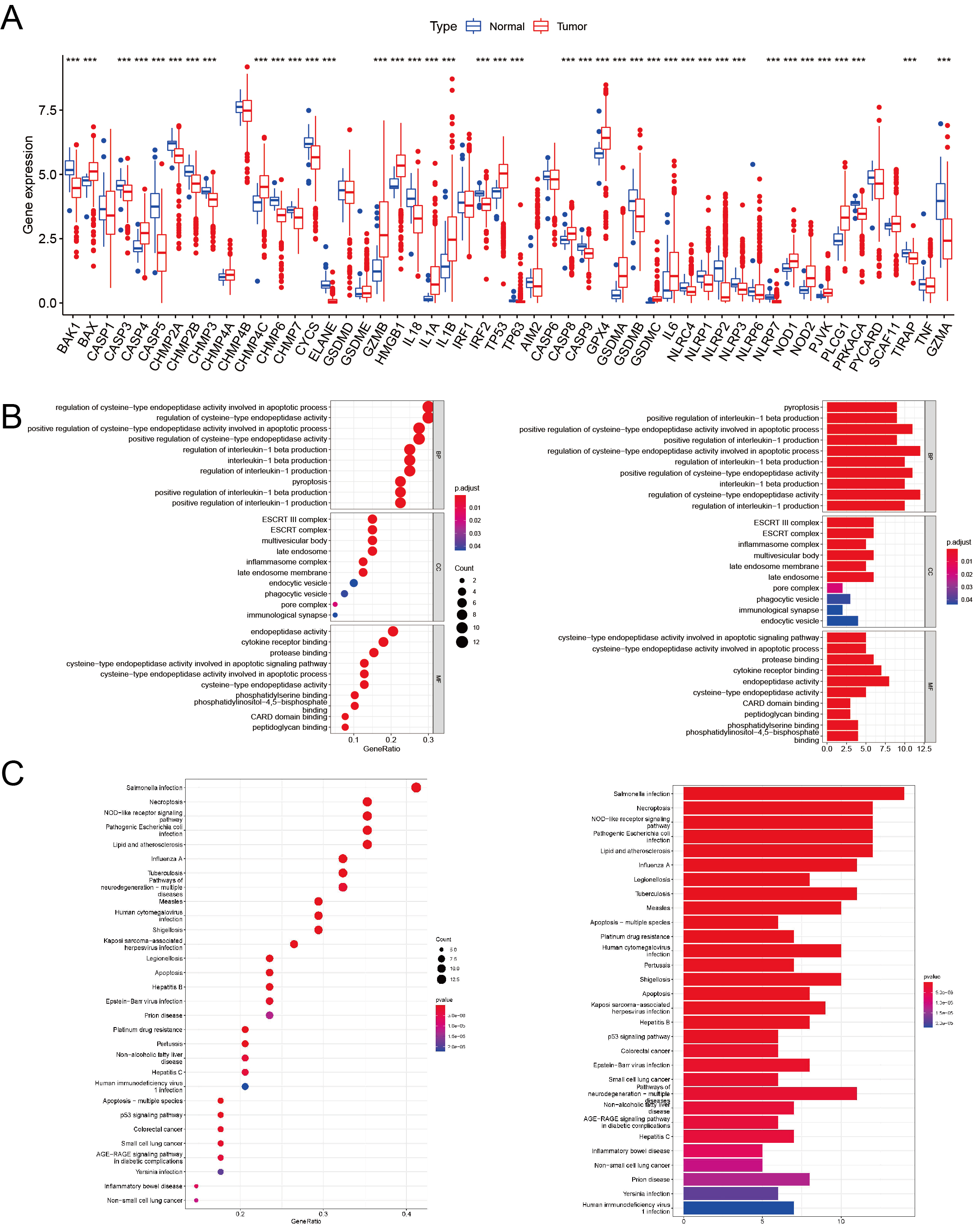

Supplement: Supplementary file 2 [file Image1.JPEG]

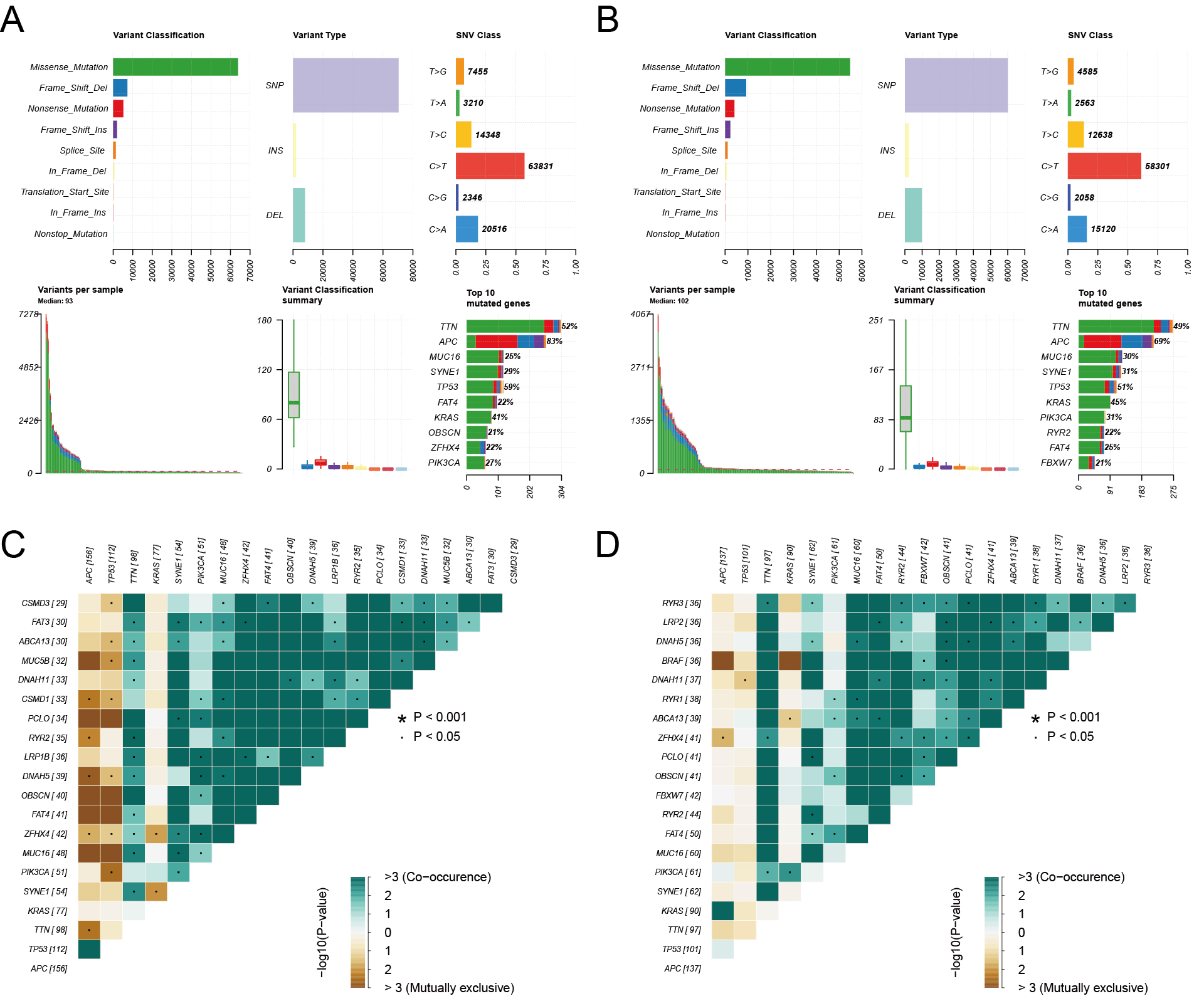

Supplement: Supplementary file 3 [file Image2.JPEG]

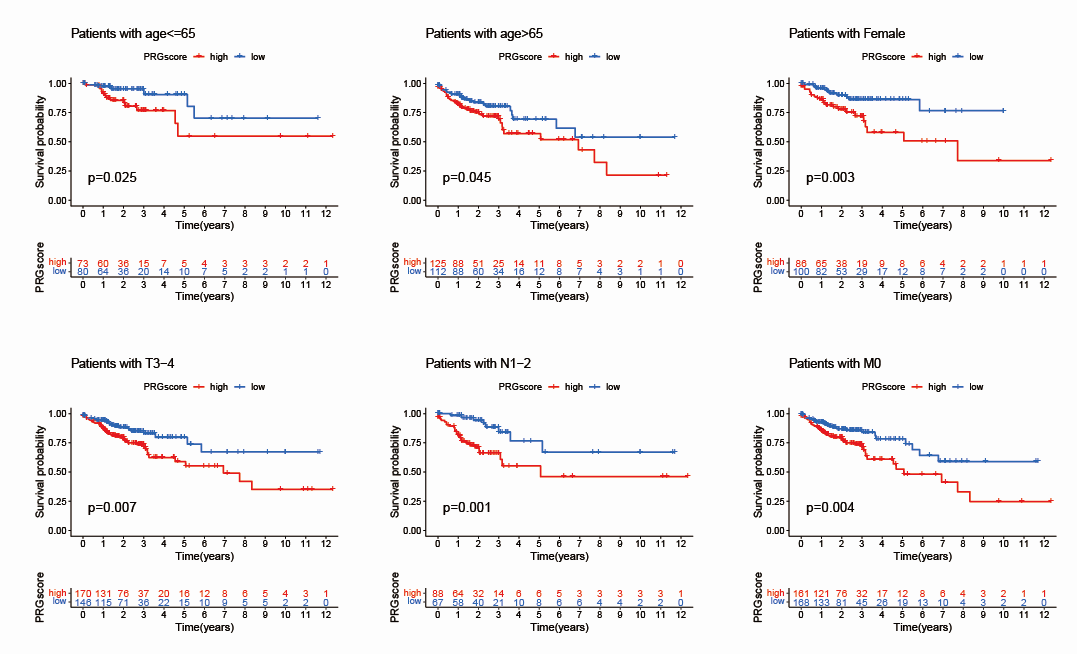

Supplement: Supplementary file 6 [file Image5.TIF]

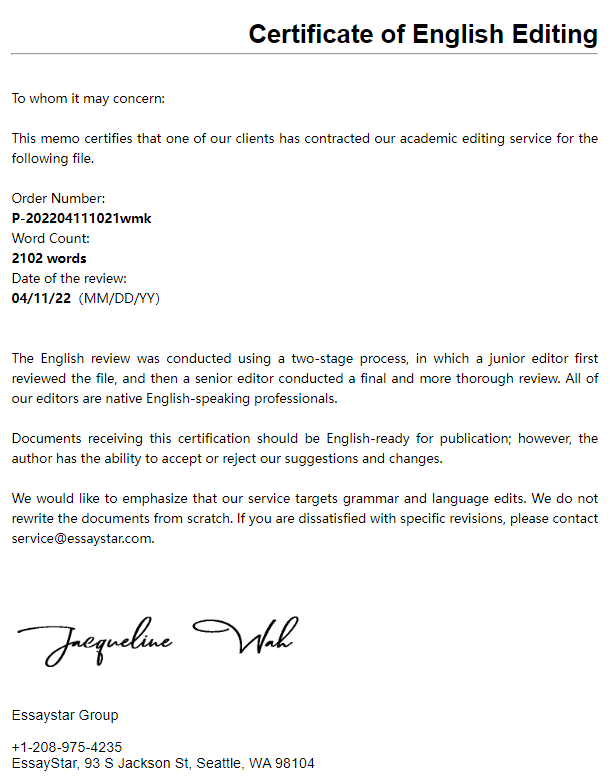

Supplement: Supplementary file 7 [file Image3.PNG]
