# Supplementary material for: Development and verification of a prognostic model for colon cancer on pyroptosis-related genes
Source: Front Genet. 2022 Sep 30;13:922055. doi: 10.3389/fgene.2022.922055 (PMC9562195; doi:10.3389/fgene.2022.922055)
Supplement: Supplementary file 4 [file Image4.PDF]

Supplementary Fig. 1 Functional analysis based on the DEGs in the TCGA cohort. A. The RNA levels of the pyroptosis-related genes are presented as boxplot between the normal and the tumour tissues. B Bubble and Barplot graph for GO enrichmen. C Bubble and Barplot graph for KEGG pathways.

Supplementary Fig. 2 Landscape of mutation profiles between high- and low-risk CC patients. (A, B) Variant classification and distribution of genetic alterations. (C, D) The interactions between the mutated genes. The darker the color, the stronger the co-occurrence.

Supplementary Fig. 3: The Kaplan–Meier overall survival curves between high- and low-risk patients with different clinical characteristics colon cancer.
