# Supplementary material for: Development and verification of a prognostic model for colon cancer on pyroptosis-related genes
Source: Front Genet. 2022 Sep 30;13:922055. doi: 10.3389/fgene.2022.922055 (PMC9562195; doi:10.3389/fgene.2022.922055)
Supplement: Supplementary file 5 [file Table2.DOCX]

Fig. 1 Expressions of the 40 differentially expressed genes in all 52 pyroptosis-related genes and the interactions among them. A Heatmap of the pyroptosis-related genes between the normal and the tumour tissues. P values were showed as: **P < 0.01; ***P < 0.001. B PPI network showing the interactions of the pyroptosis-related genes (interaction score= 0.9). C The correlation network of the pyroptosis-related genes (red line: positive correlation; blue line: negative correlation).

Fig. 2 Landscape of genetic and expression variation of PRGs in CC. A The CNV variation frequency of 33 PRG in the CC cohort. The height of the column represented the alteration frequency. B Waterfall plots of mutation information in CC. C The location of CNV alteration of PRGs on 23 chromosomes in the CC cohort.

Fig. 3 Tumour classification based on the pyroptosis-related DEGs. A 446 CC patients were grouped into two clusters according to the consensus clustering matrix (k=2). B Heatmap and the clinicopathologic characters of the two clusters classified by these DEGs. C Kaplan–Meier OS curves for the two clusters.

Fig. 4 KEGG pathway enrichment analyses between two clusters groups in the TCGA cohort.
Fig. 5 Construction of risk signature in the TCGA cohort and verification the model in the Geo cohort (GSE103479) A Univariate cox regression analysis of OS for each pyroptosis-related gene, and 54 genes with P < 0.001. B LASSO regression of the 3 OS-related genes. C Cross-validation for tuning the parameter selection in the LASSO regression. D Distribution of patients based on the risk score in the TCGA cohort. E The survival status for each patient in the TCGA cohort. F Kaplan–Meier curves for comparison of the OS between low- and high-risk groups in the TCGA cohort. G Time-dependent ROC curves for OCs in the TCGA cohort. H Distribution of patients based on the risk score in the GEO cohort. I The survival status for each patient in the GEO cohort. J Kaplan–Meier curves for comparison of the OS between low- and high-risk groups in the GEO cohort. K Time-dependent ROC curves for OCs in the GEO cohort.

Fig. 6 Landscape of mutation profiles between high- and low-risk CC patients and Evaluation of the prognostic role of PRGs signature. (A, B) Waterfall plots of mutation information in each sample. C The univariate Cox forest map of risk model score and clinical features in the TCGA cohort. D The multivariate Cox forest plot of risk model score and clinical characteristics in the TCGA cohort. E Establishment of a nomogram for 1-, 2-, and 3-year OS prediction in CC.

Fig. 7 Evaluation of the prognostic model with clinical characteristics in colon cancer. A Heatmap for the connections between clinical characteristics and the risk groups (*P < 0.05, **P < 0.01). B The distributions of the risk score in CC patients with different clinical characteristics. C Kaplan–Meier survival curves according to the mRNA expression of SLC2A3, TMPRSS11E and UPK3B in CC tissues.

Fig. 8 SLC2A3, TMPRSS11E and UPK3B activity is required for tumor cell proliferation and migration. RKO cells were treated with either a control siRNA or a siRNA targeting SLC2A3, TMPRSS11E and UPK3B. transduced cells were used for the analysis of mRNA expression by RT-qPCR and cell function. A The mRNA levels in these established cell lines were verified by RT-qPCR assay. B CCK8. C Colony formation assay. D Cell-based scratch assay

Fig.9 Immunohistochemistry (IHC) results showing protein levels of PRGs in CC and normal tissues. A IHC results of SLC2A3 in normal tissue and in CC. B IHC results of TMPRSS11E in normal tissue and in CC. C IHC results of UPK3B in normal tissue and in CC.
