# Supplementary material for: Development and verification of a prognostic model for colon cancer on pyroptosis-related genes
Source: Front Genet. 2022 Sep 30;13:922055. doi: 10.3389/fgene.2022.922055 (PMC9562195; doi:10.3389/fgene.2022.922055)
Supplement: Supplementary file 1 [file Table1.DOCX]

Dear Editor,

We would like to submit the enclosed manuscript entitled “*Development and verification of prognostic model for colon cancer on pyroptosis-related genes*”, which we wish to be considered for publication in ***frontiers in genetics.***

We believe that this manuscript will make it interesting to general readers of ***frontiers in genetics***. Pyroptosis, a novel form of programmed cell death, was found to play a dual role in tumor development and therapeutic mechanisms in recent years. In colon cacner, how *pyroptosis-related genes* interact and whether they are related to the survival time of patients remain unknown.

However, up to now, how PRGs interact and whether they are related to the survival time of patients remain unknown. Therefore, the aim of our current study was to development of a prognostic gene model in the TCGA. And this valuable information of prognosis could help us to make clinical decision, determine the strategy of surveillance, and predict the short- and long-term survival status for colon cancer patients. Additionally, our results may provide significant evidence for prognostic biomarkers and therapeutic targets in colon cancer.

This work is not under active consideration for publication, has not been accepted for publication, nor has it been published, in full or in part. This study was supported by the National Nature Science Foundation of China (No. 81871962), Project (RS2020-09) supported by Peking University People’s Hospital Scientific Research Development Fundsand and Industry-University-Research Innovation Fund in Ministry of Education of the People’s Republic of China (No. 2018A01013).None of the authors has any conflicts of interest associated with the work presented in this manuscript. All authors had access to the data and played a role in this manuscript. The corresponding author had full access to all the data in the study and had final responsibility for submission for publication.

Thank you for your attention and consideration.

Sincerely yours,

Shan Wang

Yingjiang Ye

Kewei Jiang

**Shan Wang**, MD, PhD

Department of Gastroenterological Surgery, Peking University People’s Hospital, Beijing, China

Laboratory of Surgical Oncology, Beijing Key Laboratory of Colorectal Cancer Diagnosis and Treatment Research, Peking University People’s Hospital, Beijing, China
E-mail: [shanwang60@sina.com](mailto:shanwang60@sina.com)

**Yingjiang Ye**: MD, PhD

Department of Gastroenterological Surgery, Peking University People’s Hospital, Beijing, China

Laboratory of Surgical Oncology, Beijing Key Laboratory of Colorectal Cancer Diagnosis and Treatment Research, Peking University People’s Hospital, Beijing, China
E-mail:yeyingjiang@pkuph.edu.cn

**Kewei Jian**g: MD, PhD

Department of Gastroenterological Surgery, Peking University People’s Hospital, Beijing, China

Laboratory of Surgical Oncology, Beijing Key Laboratory of Colorectal Cancer Diagnosis and Treatment Research, Peking University People’s Hospital, Beijing, China
E-mail: [jiangkewei@pkuph.edu.cn](mailto:jiangkewei@pkuph.edu.cn)
